# Macrolide Resistance and P1 Cytadhesin Genotyping of *Mycoplasma pneumoniae* during Outbreak, Canada, 2024–2025

**DOI:** 10.3201/eid3112.250872

**Published:** 2025-12

**Authors:** Zareen Fatima, Padman Jayaratne, Amjad Arrabi, Candy Rutherford, Daniela Leto, Marek Smieja, Mohammad Rubayet Hasan

**Affiliations:** McMaster University, Hamilton, Ontario, Canada (Z. Fatima, P. Jayaratne, D. Leto, M. Smieja, M.R. Hasan); Research Institute of St. Joe’s Hamilton, Hamilton (Z. Fatima, A. Arrabi, M. Smieja, M.R. Hasan); Hamilton Regional Laboratory Medicine Program, Hamilton (P. Jayaratne, C. Rutherford, D. Leto, M. Smieja, M.R. Hasan)

**Keywords:** pneumonia, Mycoplasma pneumoniae, bacteria, respiratory infections, antimicrobial resistance, macrolide resistance, P1-genotype, Canada

## Abstract

We investigated macrolide resistance and P1 genotypes of *Mycoplasma pneumoniae* during the 2024–2025 outbreak in Hamilton, Ontario, Canada. Macrolide resistance remained stable at ≈10%–20%, but significant shifts in P1 genotype distribution and resistance rates in P1 types occurred, indicating notable changes in *M. pneumoniae* molecular epidemiology in Ontario since 2011–2012.

*Mycoplasma pneumoniae* is a cause of upper and lower respiratory tract infections, particularly in children, and occurs in endemic and epidemic patterns. Although tracheobronchitis is common, pneumonia is the most clinically significant manifestation, accounting for ≈4%–8% of community-acquired bacterial pneumonias during endemic periods (*1*). Macrolides remain the primary therapy for *M. pneumoniae*, but the global rise in clinically relevant resistance is posing increasing challenges to treatment (*2*). Since COVID-19 pandemic restrictions were scaled back during 2023, *M. pneumoniae* incidence and outbreaks have increased substantially worldwide (*3*–*5*). In Ontario, Canada, a delayed but unprecedented rise in detection, reaching up to 30% positivity, has been reported since May 2024 (*6*). We assessed macrolide resistance rates and P1 cytadhesin types of *M. pneumoniae* during the 2024–2025 outbreak in Hamilton, Ontario, Canada, and compared them with strains collected before the COVID-19 pandemic.

## The Study

During January 2024–April 2025, a total of 4,297 nasopharyngeal swab (NPS) specimens from 3,717 patients were received by the Microbiology Laboratory of the Hamilton Regional Laboratory Medicine Program in Hamilton for *M. pneumoniae* detection by a laboratory-developed PCR. We screened all *M. pneumoniae*–positive samples (n = 417 after removing duplicates) for macrolide resistance by PCR genotyping and performed P1 cytadhesin typing on a randomly selected ≈25% subset of positive specimens from each month (n = 110) by amplifying the RepMP4 region of P1 cytadhesin gene (*7*) and sequencing with nanopore technology (Oxford Nanopore, https://nanoporetech.com) (Appendix). In addition to specimens received during the postpandemic period, additional *M. pneumoniae*–positive samples received during 2013–2020 were tested for macrolide resistance (n = 45) and P1 types (n = 23). We assessed statistical significance of differences in *M. pneumoniae* detection rates and macrolide resistance among different patient groups using the χ^2^ test with Yates correction to adjust for small sample size (Appendix). 

**Figure 1:**
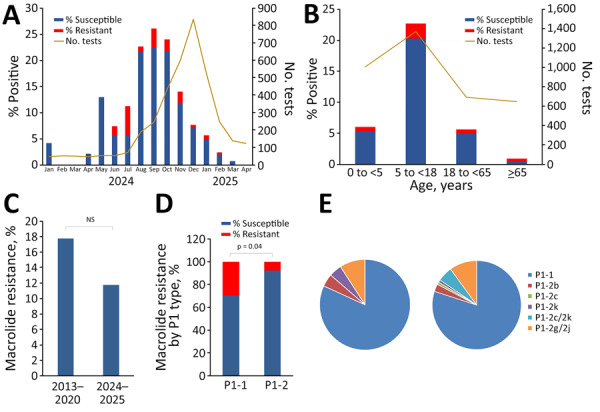
Prevalence, macrolide resistance rates, and P1 genotype distribution of *Mycoplasma pneumoniae* during 2024–2025 outbreak, Hamilton, Ontario, Canada. A) Monthly detection rates of macrolide-susceptible and -resistant *M. pneumoniae* during January 2024–April 2025. B) Detection rates of macrolide-susceptible and -resistant *M. pneumoniae* by age group. C) Comparison of macrolide resistance rates in *M. pneumoniae* before and after primary COVID-19 pandemic years. D) Macrolide resistance rates among different P1 types of *M. pneumoniae*. E) Distribution of P1-1 and P1-2 variant types among *M. pneumoniae* strains before and after primary COVID-19 pandemic years. p value was obtained from χ^2^ test with Yates correction. NS, not significant.

On average, 14.2% (381/2,680) of patients tested positive for *M. pneumoniae* in 2024, compared with 0.34% (2/576) in 2022 and 0.36% (2/555) in 2023. Since May 2024, the positivity rate gradually increased, reaching a peak of 22.5% in September 2024. After September, positivity rates steadily declined to <5% by January 2025, despite increased testing volumes through December 2024. Macrolide resistance rates varied by month and accounted for 11.8% of all positive samples during January 2024–April 2025; the highest rate of resistance (50%) was noted in July 2024 (Figure 1, panel A; Appendix Table 2). PCR genotyping identified only 1 single-nucleotide polymorphism (SNP) associated with macrolide resistance: A2063G, which is known to confer high-level macrolide resistance (erythromycin MIC >64 mg/L). This finding is consistent with a study reporting that >90% of isolates from Ontario during 2011–2012 carried the same mutation (*8*). 

As expected, *M. pneumoniae* detection rates were much higher (≈20%) in children 5–<18 years of age than in other age groups. The macrolide resistance rate for *M. pneumoniae* in patients in this age group (≈11% of all positives) was not significantly different from the rates of resistance observed in children <5 years of age or adults 18–<65 years of age (Figure 1, panel B). In contrast, 50% of *M. pneumoniae*–positive strains from patients >65 years of age were macrolide resistant, a rate that was significantly higher than in children 5–<18 years of age (p = 0.02). Although the specimen number for the >65-year-old group was low (n = 6), the higher rate of macrolide resistance in this group could be related to higher likelihood of macrolide use in elderly patients.

We next compared macrolide resistance rates in *M. pneumoniae* strains during 2013–2020 and 2024–2025 (representing COVID-19 prepandemic and postpandemic periods), which were 17.8% (prepandemic) and 11.8% (postpandemic); the difference was not statistically significant (p = 0.24) (Figure 1, panel C). By P1 typing, 89/110 (81%) *M. pneumoniae* strains belonged to the P1-1 type, and 21/110 (19.1%) belonged to the P1-2 type. The macrolide resistance rate in P1-1–type *M. pneumoniae* strains was 29.9%, significantly higher (p = 0.04) than that in P1-2–type strains (7.7%) (Figure 1, panel D). During 2013–2020, 78.3% of *M. pneumoniae* strains were the P1-1 type, compared with 81% in 2024–2025. The proportions of P1-1 and P1-2 types were not significantly different between the 2 periods (p = 0.85). Among the P1-2 type *M. pneumoniae* strains, 2k, 2b, and 2g/2j variants were detected in specimens collected during 2013–2020. There was a predominance of 2g/2j variants (50%) in both periods, but the percentage of 2c/2k variants (36.4%) increased during 2024–2025 (Figure 1, panel E).

**Figure 2:**
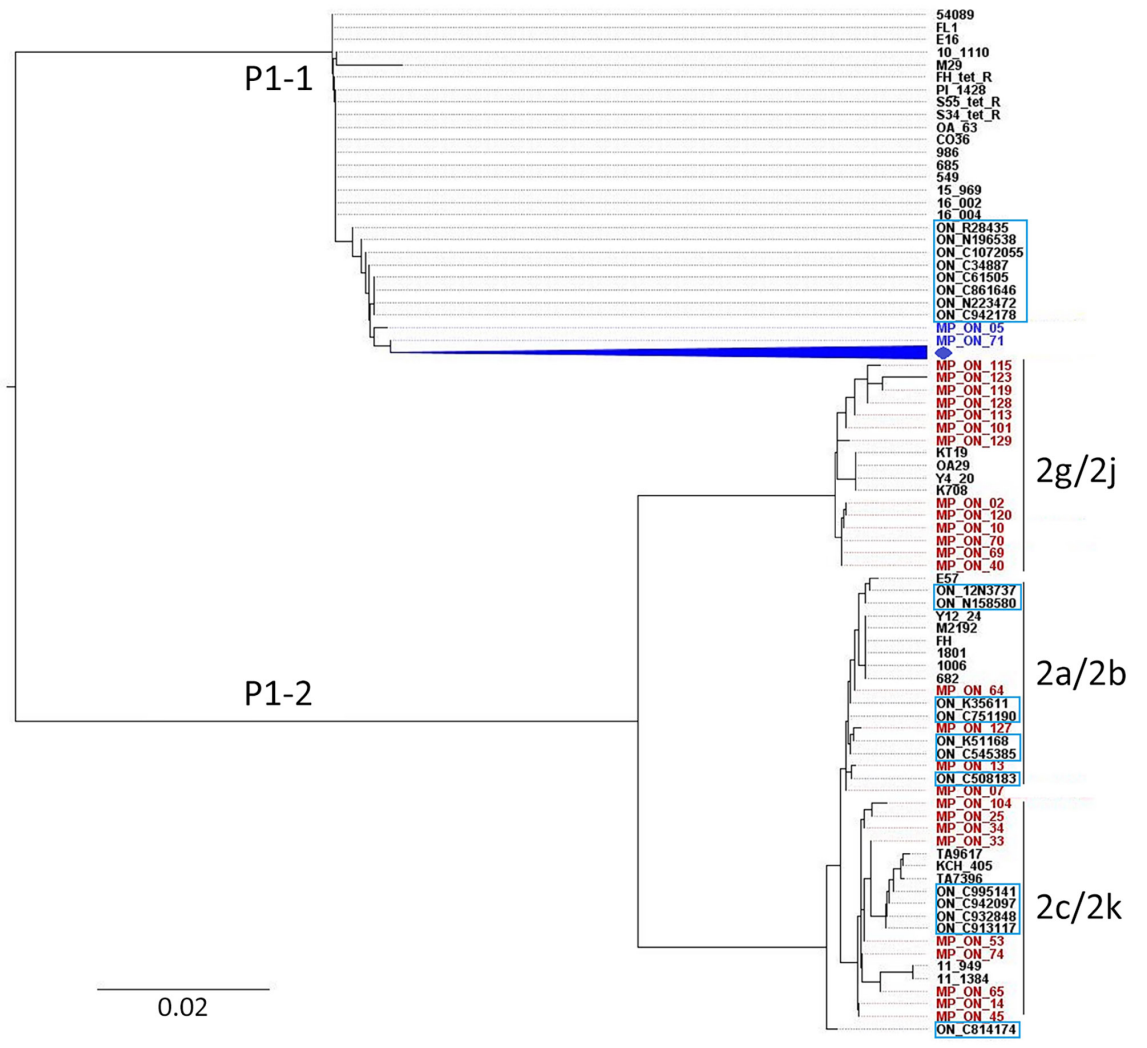
Phylogenetic analysis of *Mycoplasma pneumoniae* based on P1 cytadhesin–RepMP4 genotyping during 2024–2025 outbreak, Hamilton, Ontario, Canada. An unrooted tree was constructed using the neighbor-joining method with the Tamura-Nei model in MEGA X (https://www.megasoftware.net) using aligned sequences generated using Clustal Omega (https://www.ebi.ac.uk/jdispatcher/msa/clustalo). Strains highlighted in blue and red represent the P1-1 and P1-2 type strains assessed in this study. Strains in the light blue boxes indicate previously reported strains from Ontario during 2011–2012 (*8*). Strains shown in black represent reference RepMP4 sequences from *M. pneumoniae* obtained in other countries, representing P1 types and variants (Appendix Table 3). Blue diamond indicates remaining 104 P1-1 strains from this study.

Phylogenetic analysis of the RepMP4 region of *M. pneumoniae* strains indicated that all P1-1 type strains (95.1%), including specimens from 2017–2020 (n = 16), clustered together on a distinct branch, separate from previously described strains from Ontario reported >10 years ago (8) (Figure 2). That previous study, conducted at the Public Health Ontario Laboratory, included representative specimens from across Ontario, including submissions from the Hamilton region (S.N. Patel, Public Health Ontario, pers. comm., email, 2025 Jul 28). Only 2 strains (MP_ON_05 and MP_ON_71) from the current outbreak showed homology with previously collected strains from Ontario, suggesting the RepMP4 region of P1-1–type strains has evolved over time while circulating in Ontario. Among the P1-2 types, 4 strains (15.4%) clustered with previously reported P1-2b variants, and 1 strain appeared related to P1-2c variants reported earlier in Ontario. In addition, our study revealed that P1-2 variants 2g/2j were circulating in Ontario as early as 2013, despite not having been previously reported in the region.

The first limitation of our study is that P1 typing was performed on only a subset of samples, and variant analysis relied solely on RepMP4 sequencing. Consequently, some P1-2 variants could only be assigned to variant groups (i.e., 2g/2j and 2c/2k). Moreover, few samples from 2013–2020 were available for P1 genotyping. Nevertheless, despite stable macrolide resistance rates, our findings show a major shift in the molecular epidemiology of *M. pneumoniae* since 2011-2012. Earlier Ontario data (2011–2012) reported P1-1 made up 38.1% of strains and P1-2 61.9% of strains (*8*), whereas in our study, 81% of P1-typed strains from the 2024–2025 outbreak were P1-1 type. Furthermore, unlike the previous study, which found no association between macrolide resistance and P1 types, our study shows significantly higher rates of macrolide resistance in the P1-1 group of *M. pneumoniae*. In addition, the distribution of P1-2 variants during the postpandemic period appeared more diverse than in the prepandemic period, and the P1-2c/2k variants expanded postpandemic. Our data, however, represent only the population of the Hamilton region; regional variation elsewhere in Ontario cannot be excluded.

The percentages of P1-1 versus P1-2 types in specimens from 2013–2020 (Figure 1, panel E) differs from previous reports for 2011–2012 (8). That discrepancy reflects that most prepandemic samples in our study (22/23) were collected during 2017–2020; only 1 was from 2013. Those data also suggest that the shift from predominantly P1-2 to P1-1 types might have begun before the pandemic.

## Conclusions

Our study provides a snapshot of macrolide resistance rates and P1 genotypes of *M. pneumoniae* strains in Hamilton, Ontario, Canada, nearly a decade after the last provincial report. Macrolide resistance rates appear to have remained stable over that time, but we observed major changes in the P1 cytadhesin types of *M. pneumoniae* circulating in the Hamilton region. Clinicians and other public health professionals should be aware of those changes and their potential effects on clinical and public health management of respiratory infections caused by *M. pneumoniae* in Ontario.

Appendix Additional information about macrolide resistance and P1 cytadhesin genotyping of *Mycoplasma pneumoniae* during 2024–2025 outbreak, Hamilton, Ontario, Canada
