# Supplementary material for: Macrolide Resistance and P1 Cytadhesin Genotyping of Mycoplasma pneumoniae during Outbreak, Canada, 2024–2025
Source: Emerg Infect Dis. 2025 Dec;31(12):2316–9. doi: 10.3201/eid3112.250872 (PMC12782266; doi:10.3201/eid3112.250872)
Supplement: Appendix — Additional information about macrolide resistance and P1 cytadhesin genotyping of Mycoplasma pneumoniae during 2024–2025 outbreak, Hamilton, Ontario, Canada [file 25-0872-Techapp-s1.pdf]

Article DOI: https://doi.org/10.3201/eid3112.250872

EID cannot ensure accessibility for supplementary materials supplied by authors. Readers who have difficulty accessing supplementary content should contact the authors for assistance.

Macrolide Resistance and P1 Cytadhesin Genotyping of *Mycoplasma pneumoniae* during Outbreak, Canada, 2024–2025

Appendix

Additional methods

Specimens and study population: For the 2024–2025 period, nasopharyngeal swab (NPS) specimens were routinely collected for *Mycoplasma pneumoniae* PCR testing from patients with upper respiratory tract infections, bronchitis, and "atypical" community-acquired pneumonia. Specimens were received at the Hamilton Regional Laboratory Medicine Program (HRLMP), which provides laboratory services to four major acute care hospitals in the Hamilton region, under St. Joseph's Healthcare Hamilton and Hamilton Health Sciences, covering a catchment area of more than 2.3 million people. These hospitals include Hamilton General Hospital, Juravinski Hospital and Cancer Centre, McMaster Children's Hospital, and St. Joseph's Healthcare Hamilton. For the 2013–2020 period, randomly selected *M. pneumoniae* positive specimens saved for test validation purposes were used. The clinical indications for testing have remained unchanged since the beginning of the study period, and specimens from 2013–2020 period were collected from the same catchment area.

***Detection of Mycoplasma pneumoniae*:** Nucleic acids from nasopharyngeal swab (NPS) specimens received by HRLMP Microbiology Laboratory for *M. pneumoniae* testing were extracted using the NUCLISENS easyMAG platform (bioMérieux) according to the standard protocol. Eluates were tested using a laboratory-developed, TaqMan-based multiplex PCR assay that simultaneously detects *M. pneumoniae* targeting species-specific 16S rRNA gene sequences and determines macrolide susceptibility using primers flanking 23S rRNA gene mutation sites

associated with macrolide resistance (base positions 2063, 2064, and 2067), along with a TaqMan probe containing wild-type nucleotides. PCR was performed according to the standard operating procedure (SOP), with primers and probes at final concentrations of 0.2 µM and 0.1 µM, respectively, using the QuantiTect® Multiplex PCR NoROX Kit on a Rotor-Gene 6500 platform (Qiagen). All primer and probe sequences are listed in Appendix Table 1.

Validation of Genotyping Assay for Macrolide Resistance: Three TaqMan probes were designed to detect single nucleotide polymorphisms (SNPs) associated with macrolide resistance (A2063G/C/T, A2064G/C, and A2067G mutations in 23S rDNA) (Appendix Table 1). SNP-qPCR was performed using the Type-it® Fast SNP Probe PCR Kit (Qiagen) on a Rotor-Gene 6500 platform, with thermal cycling conditions consisting of an initial 5-minute activation step at 95°C, followed by 40 cycles of denaturation at 95°C for 15 seconds and annealing/extension at 60°C for 30 seconds. Primers and probes were used at final concentrations of 0.2 µM and 0.1 µM, respectively. The SNP-qPCR method was developed using synthetic DNA sequence blocks containing the respective nucleotide substitutions (Integrated DNA Technologies).

To validate the SNP-qPCR results, 25 samples each of susceptible and resistant genotypes identified by SNP-qPCR were subjected to Nanopore sequencing. A 1,311 bp fragment of the 23S rDNA, harboring the SNPs associated with macrolide resistance, was amplified using Q5® High-Fidelity 2X Master Mix (New England Biolabs) and sequenced using Nanopore technology. Next-generation sequencing (NGS) libraries were prepared from 200 ng of DNA using the Native Barcoding Kit 96 V14, and sequencing was performed using R10.4.1 flow cells on a GridION device (Oxford Nanopore Technologies). Raw sequencing data, after quality filtering and adaptor trimming were de novo assembled to generate consensus sequences using the *wf-amplicon* workflow in EPI2ME (https://epi2me.nanoporetech.com). Nucleotide sequence alignment with the wild type reference sequence was performed using Geneious Prime® 2025.1.2 (https://www.geneious.com) (Appendix Figure 1). Resistant genotypes detected by SNP-qPCR were 100% concordant with sequencing results. For further confirmation, an additional 10 samples (5 susceptible and 5 resistant) were amplified using MP23SF and MP23SR primers (Appendix Table 1) and sequenced by Sanger sequencing, showing 100% concordance with the genotyping results (data not shown). No cross-reactivity of

the genotyping probes was noted during the validation study. Following validation, all non-susceptible *M. pneumoniae*-positive specimens were tested by SNP-qPCR as described above.

Macrolide resistance testing: A total of 4,297 NPS specimens from 3,717 unique patients were tested, of which 423 specimens were positive for *M. pneumoniae*. To eliminate duplicates, only the results from the first encounter were retained, resulting in 417 positives for M. pneumoniae. Among the positive samples, six patients had repeat swabs collected. *M. pneumoniae* detected in specimens from five of these six patients was macrolide-susceptible and remained susceptible upon repeat specimen collection at a later date. In only one patient macrolide susceptibility changed from susceptible to resistant during the course of infection.

P1 Cytadhesin Genotyping: For P1 typing, 25% of *M. pneumoniae* specimens from each month were randomly selected for the 2024–2025 period, while 23 out of 45 samples (51.1%) from the 2013–2020 period were selected based on the availability of sufficient specimen volume. We also confirmed that all age groups were proportionally represented in the subset of specimens that were genotyped (Appendix Figure 2). A 1,137 bp fragment from the RepMP4 region of the P1 cytadhesin gene (Reference 8) was amplified using the primers listed in Appendix Table 1. Amplification, NGS library preparation, sequencing, and data analysis were performed as described in the previous section.

Appendix Table 1. Primers and probes used in this study

Test	Target gene	Primer/Probe Name	Primer/Probe Sequence (5′ to 3′)
Mycoplasma pneumoniae detection and macrolide susceptibility PCR	16S	MPF	GAGTGTGGTAGGGAGTTTTGG
		MPR	ATCCTATTTGCTCCCCACAC
		MPP	CAL Fluor Orange 560-TGTGGAGCGGTGAAATGCGTAGAT-BHQ2
	23S	MP23SF	TCCAGGTACGGGTGAAGACA
		MP23SR	GCTCCTACCTATTCTCTACATGAT
		MP23SPWT	Quasar 670-CAACGGACGGAAAGACC-BHQ2
	Lambda phage	LNFP	GCAAAAGATGAGGCCGGAGATAT
		LRP	CCTTAACTTTGCCCACCT
		Lambda Probe	FAM-ACCAATGCTGAGATAGCTGAAGAG-BHQ1
Macrolide resistance genotyping PCR	23S	MP23SF	TCCAGGTACGGGTGAAGACA
		MP23SR	GCTCCTACCTATTCTCTACATGAT
		MP23SP2063	FAM-CAACGGGACGGBAAGACCCCG-BHQ1
		MP23SP2064	Cal Fluor Orange 560-CAACGGGACGGASAGACCCCG-BHQ1
		MP23SP2067	Cal Fluor Red 610- CAACGGGACGGAAAGGCCCCG
Macrolide resistance PCR sequencing	23S	1396_F	AAAGCGTAGGCGATGGACAA
		2687_R	AACTGGAGCATAAGAGGTGTC
P1 RepMP4 PCR sequencing	RepMP4	RepMP4-O-F	TTGGATTCTCATCCTCACCGCCACC
		RepMP4-O-R	TCAACGCGGTCAATGGCGGTACGGTTGC

Appendix Table 2. Rates of macrolide resistance in *Mycoplasma pneumoniae* by month during the 2024–2025 period.

Year	Month	Total no of test	No. positive	Macrolide Susceptible No (%)	Macrolide Resistant No (%)
2024	Jan	48	2	2 (100)	0 (0)
	Feb	52	0	0 (0)	0 (0)
	Mar	51	0	0 (0)	0 (0)
	Apr	47	1	1 100)	0 (0)
	May	54	7	7 (100)	0 (0)
	Jun	54	4	3 (75)	1 (25)
	Jul	71	8	4 (50)	4 (50)
	Aug	190	43	41 (95.3)	2 (4.7)
	Sep	245	64	55 (85.9)	9 (14.1)
	Oct	433	104	94 (90.4)	10 (9.6)
	Nov	599	84	71 (84.5)	13 (15.5)
	Dec	836	64	59 (92.2)	5 (7.8)
2025	Jan	527	29	25 (86.2)	4 (13.8)
	Feb	249	6	5 (83.3)	1 (16.7)
	Mar	138	1	1 (100)	0 (0)
	Apr	124	0	0 (0)	0 (0)
	**Total**	**3718**	**417**	**368 (88.2)**	**49 (11.8)**

Appendix Table 3. Description of isolates retrieved from NCBI nucleotide database for construction of phylogeny in Figure 2.

Strain	Accession	Type	Country	Year	Source
ON-C942178	KF154743.1	1	Canada:ON	2012	Throat Swab
ON-C34887	KF154746.1	1	Canada:ON	2012	NPS
ON-C1072055	KF154745.1	1	Canada:ON	2011	Oral swab
ON-N196538	KF154747.1	1	Canada:ON	2011	NPS
ON-C61505	KF154740.1	1	Canada:ON	2012	Throat Swab
ON-C861646	KF154742.1	1	Canada:ON	2011	NPS
ON-N223472	KF154741.1	1	Canada:ON	2011	BAL
ON-R28435	KF154744.1	1	Canada:ON	2011	Throat Swab
549	CP017330.1	1	USA: Washington	1965	Clinical
10_1110	CP039787.1	1	South Korea: Seoul	2010	NPS
FH_tet_R	NZ_CP020690.1	1	People’s Republic of China: Beijing	2016	-
S34_tet_R	NZ_CP020710.1	1	People’s Republic of China: Beijing	2016	Throat Swab
S55_tet_R	NZ_CP020692.1	1	People’s Republic of China: Beijing	2015	-
685	CP017328.1	1	Denmark	1988	Clinical
986	MJIZ01000002.1	1	Kenya	1998	Clinical
54089	CP010542.1	1	USA:AL	2009	Throat Swab
CO36	MJIT01000001.1	1	USA:Colorado	2013	Clinical
E16	NZ_CP017332.1	1	Egypt	2010	-
FL1	NZ_CP017333.1	1	USA:Florida	2012	-
PI_1428	CP010538.1	1	USA	1960	Throat Swab
16_002	NZ_CP039767.1	1	Korea	2016	NPS
M29	CP008895.1	1	People’s Republic of China	2005	Throat Swab
OA_63	BSFY01000002.1	1	Japan	2020	Throat Swab
15–969	NZ_CP039769.1	1	South Korea: Seoul	2015	NPS
16–004	NZ_CP039766.1	1	South Korea: Seoul	2016	NPS
E57	CP017329.1	2a	Egypt	2009	Clinical
ON-C913117	KF154751.1	2c	Canada:ON	2011	NPS
ON-C995141	KF154752.1	2c	Canada:ON	2011	NPS
ON-C932848	KF154753.1	2c	Canada:ON	2011	Throat Swab
ON-C942097	KF154754.1	2c	Canada:ON	2011	NPS
ON-N158580	KF154755.1	2a	Canada:ON	2011	NPS
ON-12N3737	KF154756.1	2a	Canada:ON	2012	NPS
ON-C814174	KF154757.1	2b	Canada:ON	2011	NPS
682	MJIU01000002.1	2b	Denmark	-	Clinical
1801	CP017341.1	2b	USA	2000	Clinical
ON-K35611	KF154748.1	2b	Canada:ON	2011	NPS
ON-C751190	KF154749.1	2b	Canada:ON	2011	Throat Swab
ON-C508183	KF154750.1	2b	Canada:ON	2011	Throat Swab
ON-C545385	KF154758.1	2b	Canada:ON	2011	NPS
ON-K51168	KF154759.1	2b	Canada:ON	2011	NPS
1006	CP017337.1	2b	USA	1999	Clinical

Strain	Accession	Type	Country	Year	Source
M2192	CP010548.1	2b	England	1982	Respiratory
Y12_24	BSFV01000012.1	2b	Japan	2020	Throat Swab
KCH_405	NZ_AP017319.1	2c	Japan	2012	-
TA9617	NZ_AP035800.1	2c	Japan: Tokyo	2020	NPS
TA7396	NZ_AP035795.1	2c	Japan: Tokyo	2017	NPS
KT19	LC588413.1	2j	Japan:Osaka	2019	-
OA29	LC588414.1	2j	Japan:Osaka	2019	-
Y4_20	LC588412.1	2j	Japan:Saitama	2016	-
K708	LC385984.1	2g	Japan:Osaka	2016	Throat Swab
11–1384	CP039775.1	2k	South Korea: Seoul	2011	NPS
11–949	NZ_CP039777.1	2k	South Korea: Seoul	2011	NPS
FH	CP010546.1	2b	USA	1954	Sputum

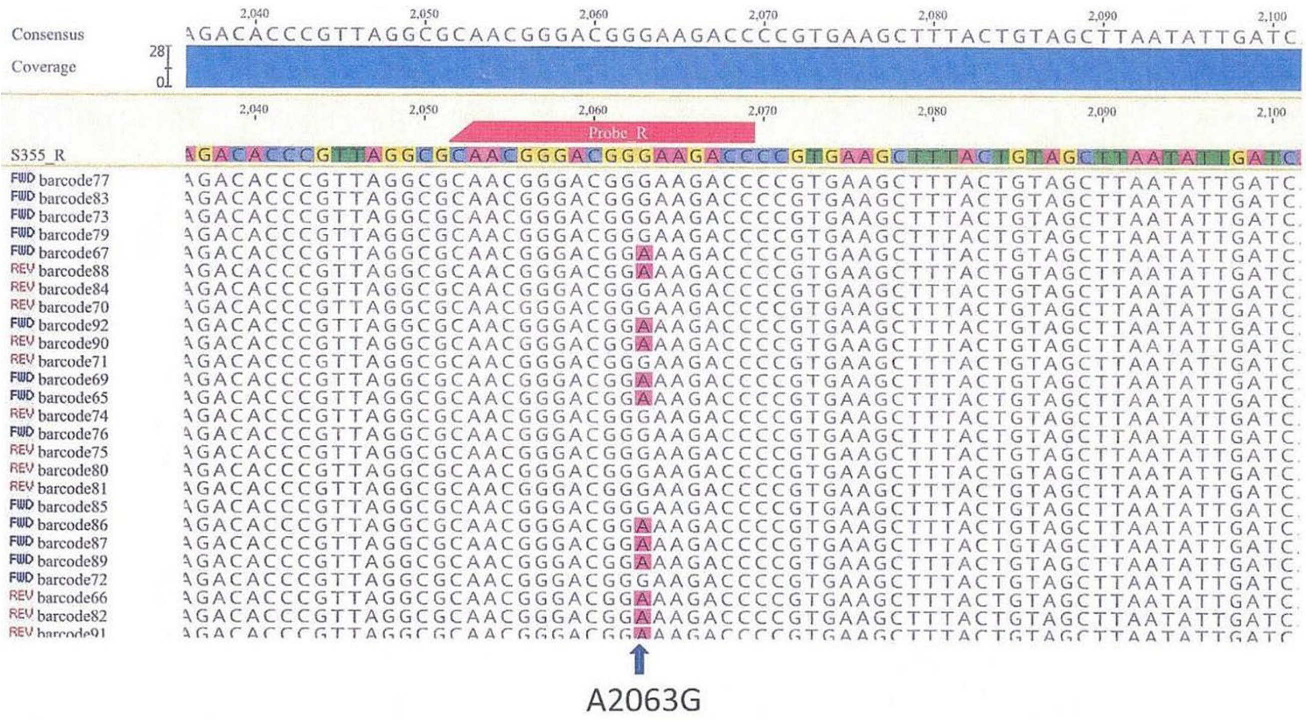

Appendix Figure 1. Nucleotide alignment showing macrolide susceptible and macrolide resistant genotypes of *M. pneumoniae.* Bases highlighted in red indicates wild-type genotype.

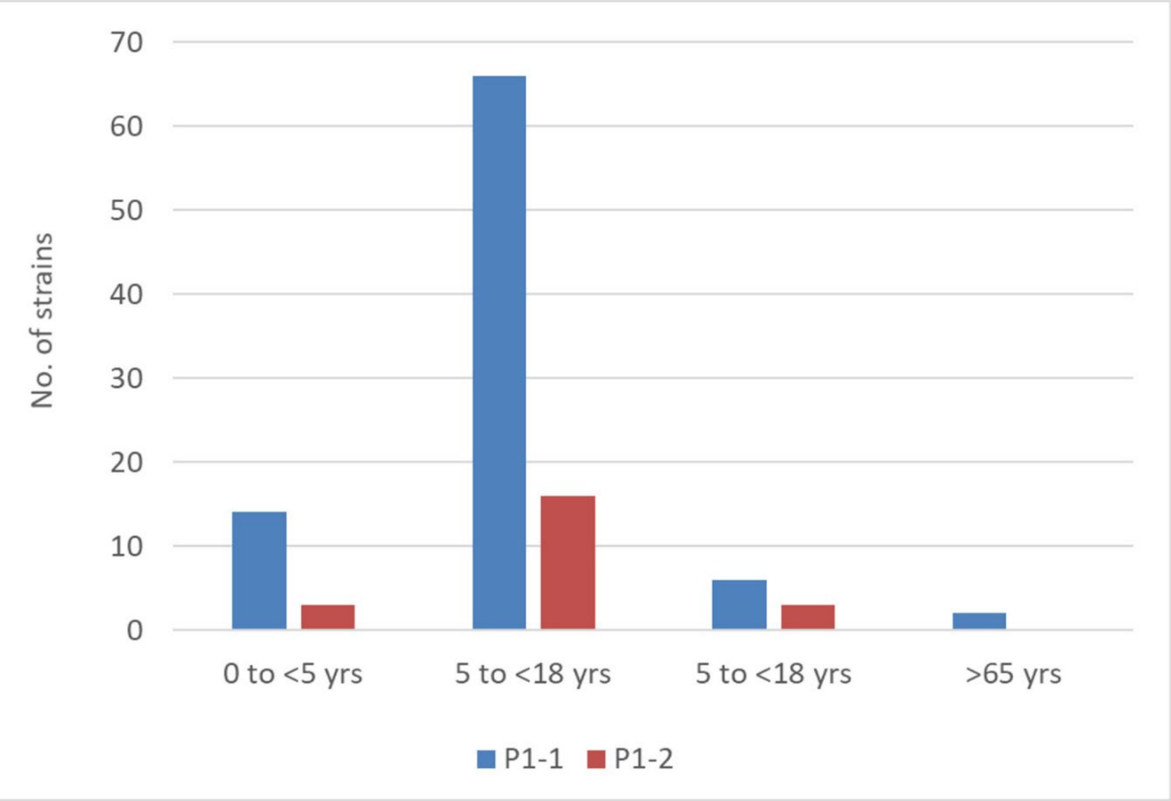

Appendix Figure 2. P1-Cytadhesin genotypes of *Mycoplasma pneumoniae* by age group.
